# Ultra high risk of psychosis on committal to a young offender prison: an unrecognised opportunity for early intervention

**DOI:** 10.1186/1471-244X-12-100

**Published:** 2012-08-03

**Authors:** Darran Flynn, Damian Smith, Luke Quirke, Stephen Monks, Harry G Kennedy

**Affiliations:** 1 National Forensic Mental Health Service, Central Mental Hospital, Dundrum, Dublin 14, Ireland; 2 Department of Psychiatry, Trinity College, Dublin, Ireland

**Keywords:** Young offenders, Ultra high risk, Psychosis, Substance misuse

## Abstract

**Background:**

The ultra high risk state for psychosis has not been studied in young offender populations. Prison populations have higher rates of psychiatric morbidity and substance use disorders. Due to the age profile of young offenders one would expect to find a high prevalence of individuals with pre-psychotic or ultra-high risk mental states for psychosis (UHR). Accordingly young offender institutions offer an opportunity for early interventions which could result in improved long term mental health, social and legal outcomes. In the course of establishing a mental health in-reach service into Ireland’s only young offender prison, we sought to estimate unmet mental health needs.

**Methods:**

Every third new committal to a young offenders prison was interviewed using the Comprehensive Assessment of At-Risk Mental States (CAARMS) to identify the Ultra High Risk (UHR) state and a structured interview for assessing drug and alcohol misuse according to DSM-IV-TR criteria, the Developmental Understanding of Drug Misuse and Dependence - Short Form (DUNDRUM-S).

**Results:**

Over a twelve month period 171 young male offenders aged 16 to 20 were assessed. Of these 39 (23%, 95% confidence interval 18% to 30%) met UHR criteria. UHR states peaked at 18 years, were associated with lower SOFAS scores for social and occupational function and were also associated with multiple substance misuse. The relationship with lower SOFAS scores persisted even when co-varying for multiple substance misuse.

**Conclusions:**

Although psychotic symptoms are common in community samples of children and adolescents, the prevalence of the UHR state in young offenders was higher than reported for community samples. The association with impaired function also suggests that this may be part of a developing disorder. Much more attention should be paid to the relationship of UHR states to substance misuse and to the health needs of young offenders.

## Background

Young offenders have greater levels of psychiatric morbidity and substance use disorders than their peers in the general population [1-4]. While at liberty, they experience multiple social disadvantages which impede their ability to seek and access appropriate treatment [5]. Young offender institutions often act as staging posts along pathways leading to mental health care, with young persons presenting via the criminal justice system rather than via community primary care routes [6]. This affords prison in-reach mental health teams a unique opportunity to identify those in need of mental health services and to provide care to a vulnerable group that may not otherwise receive it. In addition, early intervention for young offenders could result in better long term mental health, social and legal outcomes.

A recent large systematic review of adult prisoners from many different countries revealed a pooled prevalence of psychosis four times that of the general population [7]. In Ireland the six month prevalence of psychosis in adult prisons was found to be 7.6% for male remanded prisoners [8] and 2.6% for male sentenced prisoners [9]. International findings from young offender populations have reported rates of psychotic disorders lower than in adults, though ranging from 1 to 10% [1-4]. Our study is the first to report on the rates of psychosis and the Ultra High Risk mental state for psychosis (UHR) in a young offender prison. We chose to study the prevalence of UHR mental states rather than other disorders of high prevalence in prison populations such as attention deficit hyperactivity disorder (ADHD) which has received considerable attention in recent years [10]. Our rationale for focusing on UHR was based on the pragmatic aim of allocating a limited mental health resource toward identifying and treating the most serious mental disorders with the highest healthcare burden.

Reducing the duration of untreated psychosis improves the prognosis of schizophrenia in terms of symptoms, functioning and quality of life [11,12]. It is now possible to identify individuals at imminent risk of developing psychosis [13-15]. Interventions may then be used to prevent or delay the onset of psychotic disorders [16-18], although this remains an area for further research [19,20]. The Comprehensive Assessment of At-Risk Mental States (CAARMS) is a semi-structured interview which has been developed to identify individuals at ‘ultra-high risk’ of developing psychosis [21,22]. The UHR criteria combine the risk factor of age (adolescence to early adulthood) with clinical, state and trait factors identified as precursors to psychotic illness [22]. Younger age is included in the UHR criteria as it corresponds to the highest incidence for psychosis [23,24]. Young offenders are of a similar age demographic and therefore high rates of UHR mental states might be expected in young offenders.

While rates of psychosis in adult prisoners are typically in the range 2% to 4% [7], much lower rates of psychosis are reported in young offenders, typically 1% [1-4] because the onset of disorders such as schizophrenia tends to be at a later age. High rates of UHR states might however be expected in young offenders as the precursors to the high rates of psychosis in adult prisoners. The CAARMS has been validated in a help-seeking community based cohort [21], however only one previous study has used it in an adult prison setting [25]. To our knowledge we are the first group to use the CAARMS to identify the prevalence of UHR of psychosis among young offenders.

## Objectives

As part of an audit and service development project to establish a mental health in-reach service for a young offender institution, we set out to identify the prevalence of UHR states for developing psychosis and psychotic disorder in young offenders. Given the age profile (16 to 20 years) of this young offender population we hypothesised that we would find high rates of UHR states. We expected to identify a higher prevalence of psychotic disorder as compared to that of the general population, but due to their younger age we believed this would be lower than that of adult prison populations. In addition we aimed to identify the prevalence of general psychopathology and substance use disorders, hypothesising that we would find high rates of each among young offenders.

## Methods

### Study design

We interviewed every third person committed to a young offender institution over a twelve month period. Those interviewed were seen within seven days of reception. We used a semi-structured interview for background and demographic details, a semi-structured interview for substance misuse [26,27], and the CAARMS structured interview for ultra-high risk states of psychosis [21,22]. The study was approved as a clinical and service audit project by the audit, effectiveness and research ethics committee for the National Forensic Mental Health Service. All participants gave informed consent for participation.

### Setting

St Patrick's Institution is a young offender institution in Ireland with a bed capacity of 217. At the time of this study it was the only prison accepting males aged 16 to 20 in the state (population 4.6 m). All committals were screened by a prison nurse within 6 hours of reception at the prison and all were seen by a general practitioner within 24 hours who carried out an unstructured general health assessment as for any new patient presenting to primary care. One in three from a list of chronological receptions was selected for a more detailed assessment by the visiting psychiatrists (DF and DS, post-membership psychiatric registrars, equivalent to US fellows or UK ST4). If the person selected was not eligible because it was their second or subsequent committal, the next on the list was selected.

### Participants

Of the 836 committals in the study period, 78 were not eligible because they were second or subsequent committals leaving 758 eligible new committals. Every third committal eligible (n = 278) was selected for interview and 480 were not selected. Of the 278 selected, 107 were either released before they could be interviewed, declined the interview, were absent from the prison at court, released from custody or transferred to another prison and the remaining 171 were interviewed.

### Instruments

The Comprehensive Assessment of At-Risk Mental States (CAARMS) is a semi structured research diagnostic interview schedule which was developed to reliably detect the prodrome of first episode psychosis prospectively [21,22]. It has seven subscales (positive symptoms, cognitive change, emotional disturbance, negative symptoms, behavioural change, motor/physical change, and general psychopathology), each of which are scored from 0 (absent) to 6 (psychotic and severe / extreme). Each subscale scores for threshold, frequency and duration of symptoms. It takes account of the relationship between symptoms and substance use and also measures subjective level of distress caused by symptoms (0–100 scale).

To meet the symptom component of the criteria for an UHR state, only symptoms in the positive symptom subscale require assessment. This subscale is comprised of four domains: Unusual Thought Content (e.g. delusional mood and perplexity, ideas of reference, bizarre ideas), Non-Bizarre Ideas (e.g. suspiciousness, grandiose ideas, somatic ideas, nihilistic ideas, religious ideas), Perceptual Abnormalities (e.g. distortions, illusions, hallucinations), and Disorganised Speech.

The CAARMS defines three groups which outline criteria required to be diagnosed with the ultra-high risk state - 1) A “vulnerability” state in which there is a family history of psychosis in first degree relative or schizotypal personality disorder in the identified patient is present 2) “attenuated psychosis” , a pattern of psychotic symptoms which are sub-threshold in intensity or frequency for a diagnosis of psychotic disorder 3) Brief limited intermittent psychotic symptoms (BLIPS) describes a recent history of frank psychotic symptoms that resolved spontaneously without anti-psychotic medication within one week 4) a fourth category of psychotic disorder is also included in the CAARMS. This is defined as symptoms rated above threshold as described in the CAARMS and present for at least one week.

To fulfil the criteria for each of the UHR groups, quantitative evidence of functional impairment is also required as measured by a 30% drop in score on the Social and Occupational Functioning Scale (SOFAS) [28]. The SOFAS is a tethered rating scale ranging from 0 'grossly impaired' to 100 'superior functioning' and differs from the Global Assessment of Function scale (GAF) [29] in that it does not include ratings for severity of symptoms.

Inter-rater reliability was tested by jointly interviewing and independently scoring 13 individuals, Spearman rank correlation coefficient r = 0.962, p < 0.001.

Alcohol and substance use disorders were diagnosed using the short form of the Developmental Understanding of Drug Misuse and Dependence (DUNDRUM-DS) [26,27]. This instrument was developed by the authors to elicit DSM-IV diagnostic criteria for substance use disorders using a brief yet comprehensive interview. We chose not to use the alcohol and substance use modules of the Structured Clinical Interview for DSM-IV [30] for a number of reasons including the longer duration of interview required and its omission of newer substances like Mephedrone. The DUNDRUM-DS assesses the severity of use for a range of intoxicants on four levels: never used, ever used, abuse and dependence. Its validity is currently being tested in a forensic inpatient population. Joint interviewing (DS & DF) and separate scoring of ten individuals revealed good to excellent inter-rater reliability (cannabis Cohen's Kappa = 0.855 p < 0.001, mephedrone k = 0.863 p < 0.001, alcohol, ecstasy, amphetamine, cocaine, benzodiazepines and heroin all k = 1, p < 0.001).

### Data sources & measurements

All committals were identified by LQ and prison staff from a computerised list in order of time of reception at the prison and every third committal was identified. Re-committals were not eligible and were substituted by the next on the list. All participants were interviewed by one of the two psychiatrists (DF & DS).

### Study size

There is no previous research concerning the prevalence of ultra-high risk states in prisons. It is known that 3% of committals to adult prisons in Ireland have a current psychosis [31], and community help-seeking samples have shown that between 20% and 40% of ultra-high risk cases identified in community help seeking samples go on to develop psychosis [13-15,19,21,23,32,33] We inferred that with a 20% conversion rate, 15% of committals to a young offenders group might be expected to fulfil the ultra-high risk criteria. Using the Confidence Interval Analysis statistics programme [34], we calculated that a sample of 100 would detect a 15% prevalence with a 95% confidence interval of 9.3% to 23.3%.

### Measures

Cases were grouped according to whether they met criteria for each of the ultra-high risk categories or overall. Cases were also grouped according to age in one-year divisions and according to the number of substance misuse problems identified (alcohol, ecstasy, amphetamine, cocaine, mephedrone, heroin, benzodiazepines). Dwelling could only be elicited for the most recent place prior to committal, and was dichotomised into urban (any town or city) and rural. Ethnicity was similarly simplified to Caucasian Irish born, Irish Traveller [35] and other.

### Statistical methods

All data were stored anonymously and entered in SPSS-18 [36]. Where needed, added statistics were calculated using Confidence Interval Analysis [34] to derive relative risks and their confidence intervals. Age (mean 18.2, SD 1.3, range 16 to 20), SOFAS score (mean 50.4, SD 12.9, range 20 to 86) and number of substance misuse problems (mean 2.3, SD 1.8, range 0 to 7) all had the characteristics of normally distributed variables, with no outlier observations, and mean, median and mode close in each case. However number of substance misuse problems was treated as an ordinal.

Chi-squared (χ²) and relative risk statistics were calculated for associations between ordinal or categorical variables. Analysis of variance was used for normally distributed variables and categorical variables such as UHR state. Binary logistic regression using likelihood ratio was also used to examine the relationship between UHR state and co-variates.

## Results

### Participants

**Figure 1:**
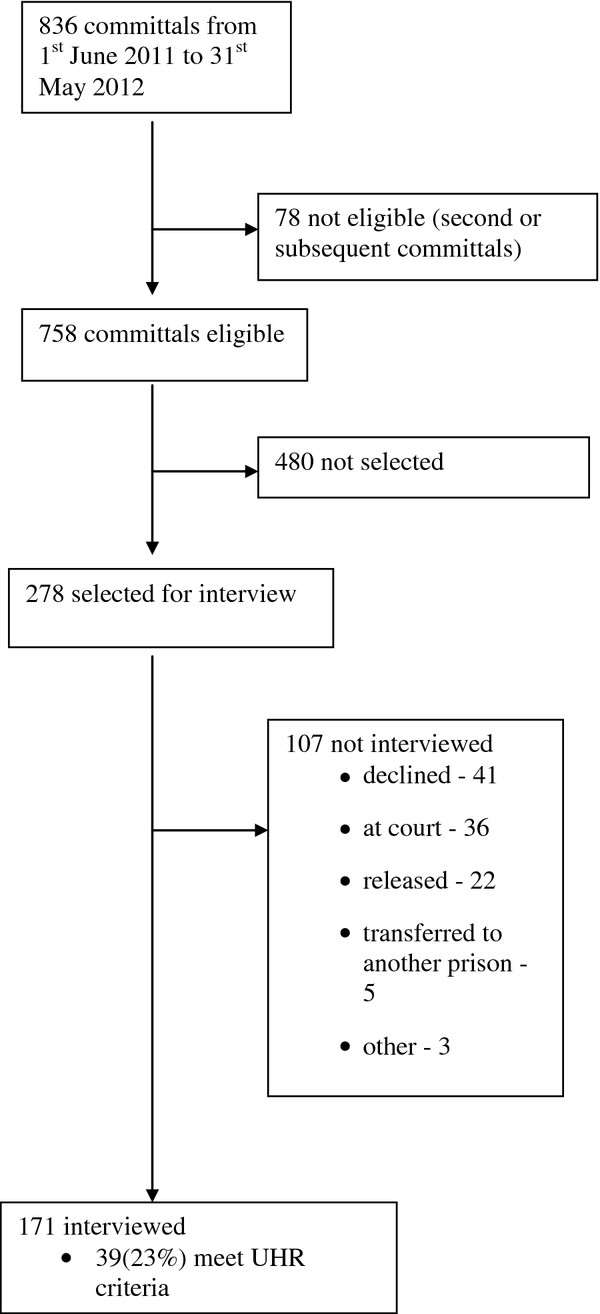
Recruitment and eligibility.

The study commenced on 1st June 2011 and the sample was completed on 31st May 2012. During this 52 week period (Figure 1) there were 836 committals of whom 758 were eligible (78 were second or subsequent committals), 278 were selected in accordance with the protocol for interviewing every third reception and 171 were interviewed. The remaining 107 included 41 who declined, 36 who were in court when called, 22 who had already been released, 5 who had already been transferred to another prison and three others.

### Bias

There was no difference in mean age between the 171 selected and interviewed (18.2 years SD 1.3), the 78 selected but not interviewed (18.4 years, SD 1.2) and the remaining 480 new committals not selected (18.4 years, SD 1.4) did not differ significantly in age (ANOVA F = 1.9, df = 2, p = 0.154). There was no significant difference in legal status (remand or sentenced χ² = 8.4, df = 2, p = 0.078) though there was a tendency for more sentenced than remands to be seen. There was no difference in geographic origin, whether from urban or rural areas (χ² = 2.0, df = 2, p = 0.364).

### Descriptive data

Of the 171 who were interviewed, the mean age was 18.2 years (SD 1.3). There were none aged 15 or less, 23 aged 16 (13.5%), 39 aged 17 (23%), 26 aged 18 (15%), 52 aged 19 (30%) and 31 aged 20 (18%). There were none older than 20. The mean time from reception to interview was 4.1 days (SD2.3, range 0 to 8). No data were missing for those included in the study and interviewed.

### Outcome data

Of the 171 interviewed, 131 (77.2%) did not meet UHR criteria. Of the 39 (22.8%) who met UHR criteria, 5 (3.5%) met the criteria for vulnerability only, 9 (5%) met criteria for vulnerability and attenuated psychosis, 19 (11.1%) met criteria for attenuated psychosis only, 1 (0.6%) met criteria for attenuated psychosis and brief limited intermittent psychosis (BLIPS), 2 (1.2%) met criteria for brief limited intermittent psychosis (BLIPS), 1 met criteria for both vulnerability and psychosis (0.6%) and 2 (1.2%) met criteria for psychosis as defined by the CAARMS - three in all met criteria for psychosis (1.8%).

UHR state was not evenly distributed according to age (Table 1), with the highest rate amongst 18 year olds (35.7%, 95% CI 20.7–54.3% compared to all others 20.2%, 95% CI 14.5–27.6%, relative risk 1.6, 95% CI 0.8–2.9, χ² =3.2, df = 1, p = 0.075).

**Table 1:** Age and ultra-high risk status

**Age (years)**	**16**	**17**	**18**	**19**	**20**	**All**
N	22	40	28	50	31	171
CAARMS positive	1(4.5%)	10 (25%)	10 (35.7%)	11 (22%)	7 (22.6%)	39(22.8%)
95% confidence interval	1.1 -21.9%	14.2 – 40.3%	20.7 – 54.3%	12.8 – 35.3%	11.4 – 40%	17.2 – 29.7%

UHR state was not related to legal status, occurring in 16 of 69 (23.2%) remand and 23 of 102 (22.5%) sentenced committals.

UHR positive state was associated with a lower mean SOFAS score 39.4(SD 6.6) v 53.7(SD 12.5) (ANOVA F = 46.5, df = 1, p < 0.001 giving a mean difference of 14.3, 95% CI 10.1 – 18.4).

UHR positive committals did not differ in age from UHR negative committals (18.1 (SD 1.4) years v 18.3 (SD 1.1), ANOVA F = 0.8. df = 1. p = 0.364.

Any substance misuse problem was present in 146 (85.4%) of those interviewed. The presence of any substance misuse problem was more common in urban dwellers (90% -v- 72% χ² = 8.1, p = 0.004) as was multiple substance misuse (χ² = 14.9, df = 7, p = 0.036). Multiple substance misuse problems were common, and number of substance misuse problems was associated with progressively lower SOFAS scores (Table 2) ANOVA F = 4.2, df 7, p < 0.001).

**Table 2:** Number of substance misuse problems and SOFAS score

**Number of substance misuse problems**	**N**	**SOFAS score**			
		**mean**	**SD**	**95% CI**	
				**Lower**	**Upper**
0	26	58	16.2	51.5	64.5
1	43	54.3	14.2	50	58.6
2	32	49.4	9.4	46	52.8
3	21	46.6	10.4	41.9	51.3
4	24	47.1	11.3	42.4	51.9
5	15	46.3	8.3	41.6	50.9
6	5	43.8	4.1	38.6	49
7	5	35.4	5.6	28.5	42.3
Total	171	50.4	13	48.5	52.4

ANOVA F = 4.2, df = 7, p < 0.001.

### Main results and other analyses

There was no significant relationship between UHR state and the presence or absence of any substance misuse problem (RR = 2.0, 95% CI 0.7 – 6.2, χ² =1.9, df = 1, P = 0.163). However there was a significant relationship between UHR status and number of substance misuse problems (range 0 to 7, χ² = 32.6, df = 7, p < 0.001; linear by linear association = 22.9, df = 1, p < 0.001) Table 3.

**Table 3:** Number of substance misuse problems and UHR status

**Number of substance misuse problems**	**CAARMS status**		**Total**
	**Negative**	**positive**	
	n (% of row)	n (% of row)	n
0	23 (89%)	3 (11%)	26
1	36 (84%)	7 (16%)	43
2	29 (91%)	3 (9%)	32
3	18 (86%)	3 (19%)	21
4	17 (71%)	7 (29%)	24
5	6 (40%)	9 (60%)	15
6	2 (40%)	3 (60%)	5
7	1 (20%)	4 (80%)	5
Total	132	39	171

χ² = 32.6, df = 7, p < 0.001.

UHR state was not related to urban–rural dwelling (χ² = 0.25, df = 1, p = 0.62) or ethnicity (χ² = 5.0, df = 5, p = 0.45).

When the effect on SOFAS score of UHR state was examined using univariate analysis of variance to co-vary for number of substance misuse problems, positive UHR state had a significant effect on SOFAS score (mean SOFAS for UHR negatives = 53.7(SD 12.5), UHR positives = 39.4 (SD 6.6), F = 28.6, df = 1, p < 0.001) and number of substance misuse problems also had a significant effect (F = 10.3, df = 1, p = 0.002).

Binary logisitic regression was used with the presence of the UHR state as dependent variable while multiple substance misuse and SOFAS scores were co-variates. Forward stepwise logistic regression with likelihood ratios yielded both co-variates as significant components of the regression equation (multiple substance misuse odds ratio = 1.478, 95% CI 1.154-1.892, p = 0.002; SOFAS score odds ratio 0.864, 95% CI 0.814-0.917, p = 0.001). This model was robust and emerged also from backward stepwise logistic regression using likelihood ratios.

## Discussion

### Key results

We have found that 22.8% (95% CI 17.7% - 30.3%) of new committals to a young offender institution met criteria for ultra-high risk of psychosis. UHR state peaked at age 18 and was associated with increasing number of substance misuse problems. UHR state was strongly associated with lower SOFAS scores, even when correcting for multiple substance misuse problems.

### Limitations

This finding may not translate into the high rates of transition to psychosis reported in community studies of help-seeking patients [13-15,19,23,32]. However the strong relationship here between UHR state and lower SOFAS scores suggests that the UHR state identified in this study is associated with meaningful impairments. This is a cross-sectional study and so cannot distinguish cause from effect. However the results are in keeping with prior research on the relationship between schizophrenia and other psychoses and risk factors such as multiple substance misuse [37] while not confirming a direct relationship with urban residence [38,39]. This study was based in a young offenders institution for males (youths) and may not extrapolate to young women. The number of women of all ages committed to prison is very small, of the order of 10% of all committals and they are received in a separate women's prison [40].

### Interpretation

New receptions in adult prisons are known to have a high rate of psychiatric morbidity [7,31] and may fall through the net of community services because of social exclusion [41] and specific lack of accessibility of community mental health services for young people [6].

Our finding of very high rates of co-morbidity between the ultra-high risk (UHR) state and multiple drug and alcohol misuse problems should be interpreted with caution. Adult offenders on reception in prison are known to have high rates of drug and alcohol problems as a norm [7,42] and comparable rates were found in young offenders groups elsewhere [2,4]. Nonetheless we have shown that multiple drug and alcohol problems carried a progressively increased risk of the UHR state. Conversely, UHR state was associated with significantly impaired SOFAS scores, even when co-varying for multiple substance misuse. There is a well-described link between cannabis use in adolescence and the later development of schizophrenia [43] though the link may be less specific for UHR states [44]. Many studies of psychotic symptoms in help-seeking populations and in random community samples of young people omit to survey for co-morbid substance misuse.

The apparent peak age of 18 for the UHR state suggests that this may be a manifestation of a developmental vulnerability period [45,46]. It may be that there is an interaction between toxic effects of drugs and alcohol and a developmental vulnerability such as neural pruning in late adolescence [45]. The stress of imprisonment may be an exacerbating factor, as may psychosis itself [46].

Urban–rural residence may have complex effects on the risk of psychosis [38,39], but in this study urban–rural residence appeared not to be a significant risk factor for the UHR state and may require further study when new census data become available.

Delusions and hallucinations have recently been described in random community samples of children and younger adolescents drawn from the same areas of Ireland as in this study [47]. Auditory hallucinations and other symptoms were present in between 21% and 23% of a community sample of 11 to 13 year olds (early adolescents), associated with a diagnosis in 57%, but present only in 7% of a community sample of 13 to 16 year olds (mid adolescents), though associated with a diagnosis in 80% [47]. Symptoms, mainly auditory hallucinations, were more common in boys. The associated diagnoses were emotional, hyperkinetic and conduct disorders, and the more disorders diagnosed, the more likely the young person was to report auditory hallucinations. Auditory hallucinations may not be predictive of psychosis particularly in the younger age group, but the 'hardening' of the association with psychiatric disorders in the mid-adolescent age group requires further research to determine the prognosis for later psychosis. The UHR state as defined by the CAARMS elicits symptoms but also includes positive family history of psychosis and brief psychotic episodes as well as sub-threshold symptoms so the UHR state described here is not directly comparable with the symptoms elicited in the study by Kelleher et al. [47]. Our population of 16 to 20 year old Irish male young offenders cannot be directly compared but our finding of the UHR state in 23% associated with impairments of function and multiple substance misuse may represent a further developmental step on the way to the emergence of a severe and enduring psychosis in some of that 23%. Help-seeking populations, community samples with symptoms and diagnosable disorders and high risk populations such as young offenders all require prospective follow-up studies to determine the prognostic significance of the UHR state. For future research, we would hypothesise that there may not be differences in prognosis between these groups once the full criteria for UHR state [21,22] are met.

This is a cross-sectional study and so cannot distinguish cause from effect. However there must now be a compelling argument for the provision of drug-free wings in prisons, particularly in young offender institutions. There is a more general question about the relationship between UHR states and substance misuse in community samples of young people. The provision of drug and alcohol awareness education may also account for the declining transition rates in recent studies of the ultra-high risk status [19,48].

## Conclusions

Young offenders institutions offer a unique opportunity to identify those who meet criteria for ultra-high risk of psychosis. While the prognostic significance of the UHR state in this group is unknown, the association with impaired functioning in itself should merit a mental health intervention, and the link with multiple substance misuse shows a need for psycho-education and motivational work in a drug-free environment while in prison. Further research is required concerning the link between the UHR state, psychosis and substance misuse in this age group.

## Competing interests

The authors declare that they have no conflicts of interest. This audit was carried out as part of the provision of a new mental health in-reach service to St Patrick's Institution. The resources were mobilised from within existing manpower and funding. The service is funded by the Health Service Executive, the publicly funded health service for Ireland.

## Authors’ contributions

DF and DS carried out the interviews and participated in data analysis. LQ coordinated the system for selecting those interviewed with SM who oversaw the clinical processes and quality of data. HGK drafted the protocol, led the analysis of data and wrote the first draft of the article. All authors participated in the drafting of the final article. All authors read and approved the final manuscript.

## Pre-publication history

The pre-publication history for this paper can be accessed here:

http://www.biomedcentral.com/1471-244X/12/100/prepub
